# Supplementary material for: Association between myopia progression and quantity of laser treatment for retinopathy of prematurity
Source: PLoS One. 2022 Dec 30;17(12):e0279898. doi: 10.1371/journal.pone.0279898 (PMC9803142; doi:10.1371/journal.pone.0279898)
Supplement: Attachment — Submitted filename: Response to Reviewers.docx [file pone.0279898.s002.docx]

Reviewer #1: The topic of this article has certain significance,which explore the impact of laser treatment on the visual develop in ROP childen. after carefuly reading the article,the following factors should be considered.

1. currently, Anti-VEGF has been partially replaced the laser treatment on ROP.so ,the advancement of this article were be weakend.

*Our finding of less myopia after treatment for anterior ROP suggests that laser may be favored over anti-VEGF if the disease is far anterior.*

2. Myopia is a multifactorial disease, which is related to genetic, environmental and other factors. Therefore, the refractive status of the parents of the children with ROP should also be taken into account in the study.

*We agree that a confounder could be the refractive status of the parents. Unfortunately, this information was not available in this retrospective study. However, we are not aware of any reason to believe that parental refractive error correlates with zone of ROP so we do not think this affected our final conclusions.*

3. Eye axis related to the aging and mopia, If the length of the ocular axial involved in the research, the conclusions are more credible.

*Post-ROP myopia is not axial myopia (see references below). Unfortunately, this information was not collected in this retrospective study.*

*15.	Wu WC, Lin RI, Shih CP, Wang NK, Chen YP, Chao AN, et al. Visual acuity, optical components, and macular abnormalities in patients with a history of retinopathy of prematurity. Ophthalmology. 2012;119(9):1907–16.*

*16.	Cook A, White S, Batterbury M, Clark D. Ocular growth and refractive error development in premature infants with or without retinopathy of prematurity. Investig Ophthalmol Vis Sci. 2008;49(12):5199–207.*

Reviewer #2: Hwang et al. retrospectively investigated myopia development in laser-treated ROP. The authors report a statistical correlation between the number of applied laser spots and the degree of myopia. They conclude that higher myopia develops in infants that received more laser treatment. This study is interesting and clinically relevant. The following issues should be addressed before consideration for publication.

Major comments

1. CRYO-ROP demonstrated a strong correlation between severity of ROP and development of myopia, independent of treatment (Quinn 1992). The more posterior the disease, the higher was the risk of myopia development. As mentioned on page 8, there is similar data from ETROP. The more severe and the more posterior the disease, however, the more likely it also needs treatment.

This means that laser treatment (or the extent thereof) may just be a confounding factor in this scenario: Myopia development correlates with ROP severity, and only because more severe/more posterior disease requires more laser treatment, there appears to be a relationship between those latter two.

The only way to proof or disproof that retinal ablative treatment in ROP is indeed influencing myopia development is a randomized trial design. CRYO-ROP randomized one eye per infant to cryotherapy and the other to observation and did not find a statistical difference in subsequent myopia development in pairwise comparison (Quinn 2001). This provides strong evidence that myopia in ROP develops independent of retinal ablative treatment.

Today, clinicians in ROP treatment are faced with the challenging decision between laser and anti-VEGF. Laser has several advantages over anti-VEGF including dramatically shorter follow-up time, no risk of systemic side effects, and no endophthalmitis. To facilitate informed decision making, the non-evidence-based impression that laser causes myopia in ROP should be avoided.

These issues should be incorporated into the manuscript’s abstract and discussion. When stating that more laser is associated with more myopia, it should be clarified that more laser is a surrogate marker for more posterior/more severe disease.

*We appreciate this reviewer's perspective that the greater myopia may be due to more posterior location of active ROP disease. We incorporated this concept into the abstract, introduction and discussion.*

*The background section of the abstract now reads:*

*Previous studies found that infants with retinopathy of prematurity (ROP) who were treated for more posterior disease with a greater number of laser spots developed higher myopia. These studies included multiple physicians with variations in laser density. In treatments by a single physician, laser spot count is a better surrogate for area of avascular retina and anterior-posterior location of disease, so that the relationship with myopia can be better assessed.*

*The second paragraph of the introduction now reads:*

*Myopia occurs after ROP treatment, and the risk of myopia is also elevated in spontaneously regressed ROP [3,4]. The risk of myopia is higher after spontaneous regression of posterior disease (i.e. zone 2) compared to anterior disease (i.e. zone 3) [5]. The CRYO-ROP study found more high myopia in treated compared to control eyes, although there were more eyes that could not be refracted in the control group [6]. In the subgroup of subjects that could be refracted in both eyes, there was no difference in myopia rates between treated and control eyes [6]. Anterior-posterior location of active ROP disease correlates with myopia, as well as the extent of final vascularization after spontaneous regression [7]. During laser treatment for ROP, the entire area of avascular retina is ablated to reduce the drive for neovascularization. Posterior location of ROP, and thereby a larger area of avascular retina ablated during laser treatment, may correlate with a greater risk of myopia [8–12]. Precisely measuring the anterior-posterior location or the area of retina treated are difficult.*

*The first paragraph of the discussion now reads:*

*In subjects treated with laser for ROP, we found that more posterior disease and a larger area of treated retina corresponded to a greater degree of myopia over long term follow up. Since a single provider performed all of the laser treatments in a standard fashion, we were able to use the number of laser spots as a surrogate for the anterior-posterior location of disease and the area of treated retina. We hypothesized that eyes with more posterior disease would develop greater myopia.*

Minor comments

2. According to US and international guidelines, zone III ROP does not normally require treatment. What was the rational for treating 42 eyes with zone III ROP in this study?

*Since there are no guidelines regarding whether to treat or observe ROP in zone III, practice patterns vary. Zone III subjects were treated to reduce the need for intensive follow up with scleral depressed exams. Our inclusion of treatments for ROP in zone III actually strengthen our evaluation of the correlation between spot counts and myopia by providing greater variation in spot counts, since laser in zone III is to a smaller area that requires fewer laser spots. Our data provide evidence that treatment in zone III is unlikely to create myopia.*

3. Page 3, line 53: Myopia secondary to ROP is usually not due to increased axial length and thus not resulting in myopic macular degeneration. Therefore, what is meant by “retinal disease” caused by myopia secondary to ROP? The cited reference #3 does not seem to provide supporting evidence or clarification for this.

*This sentence was removed.*
