# Supplementary material for: Association between myopia progression and quantity of laser treatment for retinopathy of prematurity
Source: PLoS One. 2022 Dec 30;17(12):e0279898. doi: 10.1371/journal.pone.0279898 (PMC9803142; doi:10.1371/journal.pone.0279898)
Supplement: Attachment — Submitted filename: PONE-D-22-09529_comments.docx [file pone.0279898.s003.docx]

The authors retrospective studied the ROP-treated infants and found the correlation between myopia and the area of avascular retina treated rather than spot density. However, there are some questions as below.

1. Line 50~53, please add some references for this description.

2. Line 123, please keep only one ‘.’ In the end of ‘variable’

3. Line 149~152, as you described, ‘32 of eyes…’, you’d better discuss these results in the discussion.

4. In ‘Discussion’, it would more interesting if you discussed some mechanisms of the area of avascular retina contributed to the myopia beyond your hypothesis in line 163~165.
