# Supplementary material for: Association between myopia progression and quantity of laser treatment for retinopathy of prematurity
Source: PLoS One. 2022 Dec 30;17(12):e0279898. doi: 10.1371/journal.pone.0279898 (PMC9803142; doi:10.1371/journal.pone.0279898)
Supplement: Attachment — Submitted filename: reviewer comments.docx [file pone.0279898.s004.docx]

**Response to Reviewers**

PONE-D-22-09529R1

Title: Association between myopia progression and quantity of laser treatment for retinopathy of prematurity

**Journal Requirements:**

Please review your reference list to ensure that it is complete and correct. If you have cited papers that have been retracted, please include the rationale for doing so in the manuscript text, or remove these references and replace them with relevant current references. Any changes to the reference list should be mentioned in the rebuttal letter that accompanies your revised manuscript. If you need to cite a retracted article, indicate the article’s retracted status in the References list and also include a citation and full reference for the retraction notice.

*Response: We reviewed our references list and found it to be complete and correct. We have not included any retracted papers.*

**Additional Editor Comments :**

Please see reviewers' comments and revise the MS accordingly before we accept it.

*We have revised the manuscript according to the reviewers' comments.*

**Reviewer #3: The authors retrospective studied the ROP-treated infants and found the correlation between myopia and the area of avascular retina treated rather than spot density. However, there are some questions as below.**

1. Line 50~53, please add some references for this description.

*Response: added an additional reference in line 51 to:*

*Dikopf MS, Machen LA, Hallak JA, Chau FY, Kassem IS. Zone of retinal vascularization and refractive error in premature eyes with and without spontaneously regressed retinopathy of prematurity. Journal of AAPOS. 2019;23(4):211.e1-6.*

2. Line 123, please keep only one ‘.’ In the end of ‘variable’

*Response: the double period has been corrected to a single period at the end of line 128.*

3. Line 149~152, as you described, ‘32 of eyes…’, you’d better discuss these results in the discussion.

*Response: We added a sentence to the discussion, lines 177-178, "we found that in 32 of 40 eyes (80%), the refractive error category did not change between 18 months and the end of the follow up period (average of 37 months)."*

4. In ‘Discussion’, it would more interesting if you discussed some mechanisms of the area of avascular retina contributed to the myopia beyond your hypothesis in line 163~165.

*Response: I edited the discussion (lines 185-191) to read: Primate studies have indicated that defocus over the peripheral retina leads to local scleral changes in axial myopia, but does not alter anterior segment anatomy [18]. The role of the peripheral retina in myopia of ROP is suggested by data from studies including ours correlating a greater area of healthy peripheral retina with less myopia, but the mechanisms are likely to differ from that of axial myopia. Alternate experimental models that mimic the anterior segment changes seen in premature infants are needed.*
